# Exploring Perceived and Objective Measures of the Neighborhood Environment and Associations with Physical Activity among Adults: A Review and a Meta-Analytic Structural Equation Model

**DOI:** 10.3390/ijerph19052575

**Published:** 2022-02-23

**Authors:** Elisa Menardo, Stefano De Dominicis, Margherita Pasini

**Affiliations:** 1 Department of Human Sciences, University of Verona, 37129 Verona, Italy; margherita.pasini@univr.it; 2 Coaching Psychology Unit, Department of Nutrition, Exercise and Sports, University of Copenhagen, 2200 Copenhagen, Denmark; sdd@nexs.ku.dk

**Keywords:** physical activity, physical environment, meta-analytic structural equation model

## Abstract

There is an urgent need to understand factors that promote physical activities (PA) because it is one of the modifiable risk factors for global mortality. None of the previous reviews considered both perceived and objective characteristics of the same environment. The first aim was to review the literature on studies investigating the relationship between PA behavior of adults and perceived and objective physical environment measures. The second aim was to verify the potential mediational role of the perceived measure in the relationship between the objective measure of the environment and PA using meta-analytic SEM. Only 15 studies reported a relationship between PA and both environmental measures. One of the most studied characteristics of the physical environment is the accessibility to recreational/PA facilities. Both objective and subjective measures of accessibility to PA facilities are associated with PA. Meta-SEM results suggest a significant effect of the objective accessibility to facilities on PA behavior (β = 0.15) and on the perceived measure (β = 0.10), but the indirect effect was not significant. No significant effect was found for the perceived measure on PA, suggesting that individuals’ level of awareness about their environments may have played a role. This prompts a need to create awareness campaigns.

## 1. Introduction

Promoting regular physical activity or exercise (PA) by adults or children is a public health priority [1]. The most recent WHO guidelines [2] reaffirm that regular physical activity has critical importance for cognitive outcomes (e.g., memory) and mental (symptoms of anxiety and depression) [3] and physical (e.g., adiposity) health. It also improves bone and cardiometabolic health in children and reduces the risk of falls and fall-related injuries (e.g., fractures of the hip or vertebrae), osteoporosis, and adverse psychosocial outcomes in older adults [4]. Moreover, physical inactivity is the fourth leading cause of death worldwide [1] and a fundamental risk factor for all-cause and cause-specific mortality [5] and the incidence of non-communicable diseases such as cancer [6], cardiovascular disease [7], hypertension [8], and type 2 diabetes [9]. Disparities in the distribution of PA among countries worldwide are also an important issue to consider. Indeed, the “inequality index” concerning PA could be a better predictor of obesity than the amount of PA itself [10].

According to the WHO definition [11], PA means any body movement produced by skeletal muscles that involve energy expenditure, including activities carried out by working, playing, carrying out household chores, traveling, and engaging in recreational activities. The term “physical activity” should not be confused with the term “exercise”, a subcategory of physical activity characterized by being planned, structured, repetitive, and aimed at improving or maintaining one or more aspects of physical fitness. Any physical activity should be practiced in sessions of at least 10 min in duration to be beneficial for cardiorespiratory health. Both moderate intensity and vigorous physical activity bring health benefits. The WHO recommends that adults should perform at least 150–300 min of moderate-intensity aerobic physical activity, or at least 75–150 min of vigorous-intensity aerobic physical activity, or an equivalent combination of moderate-intensity and vigorous-intensity activity throughout the week for substantial health benefits [2,4]. Evidence suggests that the association between the quantity of physical activity and some diseases (e.g., cardiovascular disease mortality, cancer, and diabetes) is curvilinear: any activity is better than no activity (Bull et al., 2020; Fletcher et al., 2018).

Recent studies suggest that about 30% of adults do not meet the WHO recommendations for PA [4,12]. Moreover, compared to previous reports [13,14,15], no significant change was observed in PA levels [13], comparing the 2016 situation with the 2001 one. Since PA is one of the “easy” modifiable risk factors for global mortality, there is an urgent need to promote physical activity [4]. This goal could be reached only by understanding the factors that push people to be active or, on the contrary, slow down physical activity [16]. 

Research about PA determinants has increased in the past 40 years [17]. In Bauman’s review [17], five categories of PA determinants were identified: individual (psychological and biological), interpersonal (social support, cultural norms, and practices), environment (social, built, and natural), regional or national policy (e.g., transport systems, urban planning, and architecture; national PA plan), and global (e.g., economic development, media, marketing). Individual and interpersonal factors play a significant role in determining childhood PA, environmental and regional/national factors for adults, and global factors for the elderly [17]. Recent systematic reviews highlighted that personal and environmental changes are needed to improve PA [1,18]. The environment could interact at different levels with the intention to be active. Based on Bronfenbrenner’s ecological model [19], the social-ecologic models of PA promotion assumed that PA’s rise results from a reciprocal interaction between the individual’s biology and the environment [18,20,21]. People’s characteristics (age, gender) and individual psychological processes (e.g., locus of control, self-efficacy, self-determinism) interact with environmental features to determine behaviors [1]. 

However, the majority of the studies focused on individual-level factors [17,22] such as age, sex, health status, self-efficacy, outcome expectation, and intention to exercise [22,23,24], even if different systematic reviews or meta-analyses support positive relations between PA and different environmental characteristics [22,25,26,27,28]. The characteristics of the physical environments most studied and that have received support as PA determinants are those featuring paths/trails, park/open space, aesthetics, and accessibility to recreational/PA facilities [22,28]. 

Nevertheless, review and meta-analysis did not always show the same conclusions or evidence. For example, Choi et al. [22], reviewing reviews, reported that accessibility to PA facilities was reported as positively associated with PA only in 5 out of 15 reviews, while the remaining 10 reviews reported inconclusive results [22]. Similarly, sidewalks and aesthetics were found to be positively correlated to PA in only 3 and 4, respectively, out of 14 reviews. The discrepancy in results could probably be explained by the methodological differences between studies, such as the sample characteristics, covariates and moderators (e.g., self-selection), PA assessment (self-report vs. objective), and environment’s measure types (perceived vs. objective). For example, different environment characteristics (e.g., recreation facilities and locations, transportation environment, and aesthetic) were related to total PA in adults, whereas no environmental characteristic correlates with elderly PA [17]. Environmental determinants also depend on which PA (i.e., walking, moderate PA, and vigorous PA) and for which purpose (i.e., transportation, occupational, and leisure) was measured. For example, walking for transportation was associated with land use mix and distance to non-residential destinations, whereas recreational walking was not [29]. Perceived environmental characteristics are biased by the multiple spatial definitions for the neighborhood [27]. In some studies, it is clearly indicated as a 10 minute walk [30,31] or 2 km around the home [32]. In contrast, other studies do not provide indications, leaving participants free to respond according to their perception [33,34,35]. This difference could also explain the discrepancy among results. 

Moreover, from the literature review, it emerged that individual choice to be or not to be active may depend on both objective (i.e., number of green areas) or subjective environmental characteristics (i.e., level of greenery or restorativeness perceived) [36]. The difference between the perceived and the objective measure of the environment’s characteristics is likely to increase the inconsistency of findings across studies and reviews [28]. Few studies have investigated both measure types of the same environmental characteristics [37], and the results are not homogeneous. Some studies suggest that perception and objective measures may influence PA differently, with the first being more associated with PA than the latest [38,39,40]. The meta-analysis of Barnett [41] highlighted differences regarding the environmental characteristics investigated. The perceived park/open space measures were significantly correlated to PA, whereas the objective measures were not. On the contrary, objective measures of greenery/aesthetics, walk/bike facilities, walk/bike barriers were significantly correlated to PA, whereas perceived measures were not [41]. In another meta-analysis, no differences were found between the two environment measure types associated with leisure-time walking [27]. 

Moreover, there is insufficient information to draw a conclusion about the relationship between objective and subjective measures of the environment. Knowing the correspondence and the relationship between objectively and subjectively assessed environments is fundamental because researchers usually use perception [32]. Associations would be missed or erroneously identified if these perceptions were inaccurate or there was a systematic bias in perception reporting [32]. Some studies suggest a low correlation between objective and subjective measures of environment characteristics [32,42,43,44]. Some authors suggest that the relationship between the objective environment’s characteristics and PA could be moderate (or mediated) by how those characteristics are perceived [28], [45]. However, previous meta-analyses [27,41] compare only studies using objective or perceived environmental characteristics associated with PA behaviors in the elderly. No study has systematically investigated the association between objective environmental characteristics and their perception in adults.

For this reason, the first aim of the present paper is to review the literature about studies that have investigated the relationship between PA behavior of adults and the environment using both perceived and objective measures of the same environment’s characteristics. Unlike previous meta-analyses, our study focuses on physical environmental characteristics that can be modified to increase the likelihood of physical activity. Moreover, crime was excluded from our study because, although it influences PA [46] and depends on physical environmental characteristics (e.g., poor light illumination or environmental degradation), it is a characteristic of the social environment and not of the physical environment [47]. Subsequently, we verify the potential mediational role of the perceived measure in the relationship between an objective measure of the environment and PA using meta-analytic structural equation model techniques.

## 2. Methods

### 2.1. Literature Analysis and Inclusion Criteria

The literature search was conducted in December 2019 using Web of Science Core Collections, PubMed, and psycINFO databases. The search strategy included a combination of terms for PA (physical activity, walk, active travel, sport, exercise) and neighborhood environment. To be included in this review, the studies had to meet the following inclusion criteria:

- Participants: Healthy persons between the age of 18–65 years. When the age range was more extensive, including adolescents or the elderly, the study was included if the mean (or median) age fall in the chosen range. Studies on obese people and pregnant women were excluded.
- Articles: Peer-reviewed in English articles.
- Outcomes: Subjective physical activities. All types of physical activities excluding gardening, climbing stairs, passive exercise, and dog walking.
- Environment: outdoor spaces. Excluded indoor space (e.g., work, home, gym). The same physical characteristics of the environment must have been investigated using (at least) one objective measure and (at least) one perceived (self-report) measure. As objective measures, we included measures based on the Geographical Information System (GIS), Google Street View, mathematical formula, or national archives/instrument. The systematic social observation was excluded.
- Environmental characteristics: physical features (e.g., trees along the streets, PA facilities, bike lines, residential density). Environmental characteristics that humans could not change (e.g., weather conditions such as atmospheric precipitation or temperature) were excluded. Socio-economic (e.g., household income of neighborhood) and social (e.g., crime) variables were also excluded. As perceived measured, we included only measures comparable with objective measures, i.e., the presence of a continuous bike line, number of PA facilities, the distance between intersections and route selection.

### 2.2. Study Selection

The study selection occurred in two phases. First, titles and abstracts of studies were screened for relevance. Second, the full text of articles with relevant abstracts was consulted to determine eligibility. Where more than one paper used the same dataset or survey to report on the same type of walking (leisure, travel, or total), data from more recent survey years was chosen.

### 2.3. Data Analysis

We included studies that reported sufficient details for the calculation of correlation between (1) PA and objective measure of the availability of PA facilities, (2) PA and perceived measure of the availability of PA facilities, or (3) objective and perceived measure of the availability of PA facilities. If other summary statistics were reported or a study had insufficient information to calculate the effect size, the corresponding author was contacted and asked to provide the correlation. If authors did not answer, available data (i.e., descriptives, odds ratio, or beta) were used to compute the correlation. The odds ratio and beta coefficients were transformed in correlation [48].

First, we used metafor r’s package (version 2.4-0) to run two separate meta-analyses to analyze the average effect size for the correlation of PA behaviors with the objective and perceived availability of PA facilities. Cochran’s heterogeneity statistic (Q) was used to investigate heterogeneity [49]. Due to the limited power of Q in identifying heterogeneity in the meta-analysis [50], *p* < 0.10 is considered significant. The results of the heterogeneity test are helpful to choose between a fixed or random-effect model appropriately. We expected significant results that mean that studies are heterogeneous. Publication bias was verified using the trim-and-fill approach [51], which estimates the number of missing studies using a non-parametric method. Correlations of single studies were transformed in Fisher’s z using the following formula: z = 0.5 × ln (1 + r)⁄(1 − r). Th estimated average effect size was then re-transformed in r using the formula: r = e^2 z^ − 1⁄e^2 z^ + 1 [52].

Then, to verify the hypothesis that the perceived availability of PA facilities mediates the effect of objective availability on PA behaviors, we used the correlation-based two-step SEM (TTSEM) proposed by Cheung and Chan [53,54]. To perform the analysis, we used the metaSEM package of R [55]. Correlation-based TTSE is appropriate when the model is just identified and has many advantages. In the first step, missing correlation coefficients are easily handled, whereas in the second step, structural models can be tested [56]. In the first step, we used a random-effect model to synthesize correlation matrices because it is more appropriate when primary studies are independently conducted with different populations and measures. So, it is not reasonable to assume the homogeneity of correlation matrices [55,57]. A pooled correlation matrix was created, weighting the variables with the sample size of each study [53]. The parameters are estimated using the weight matrix generated by the weighted least squares (WLS) method. In the second step, the estimated correlation matrix and its asymptotic covariance matrix are used to fit the structural models [56]. We estimated a saturated model (all variables are associated with each other), so the model fit was unavailable. The Sobel test [58] using an unstandardized regression coefficient and standard errors was run to determine the significance of the indirect pathway.

## 3. Results

**Figure 1:**
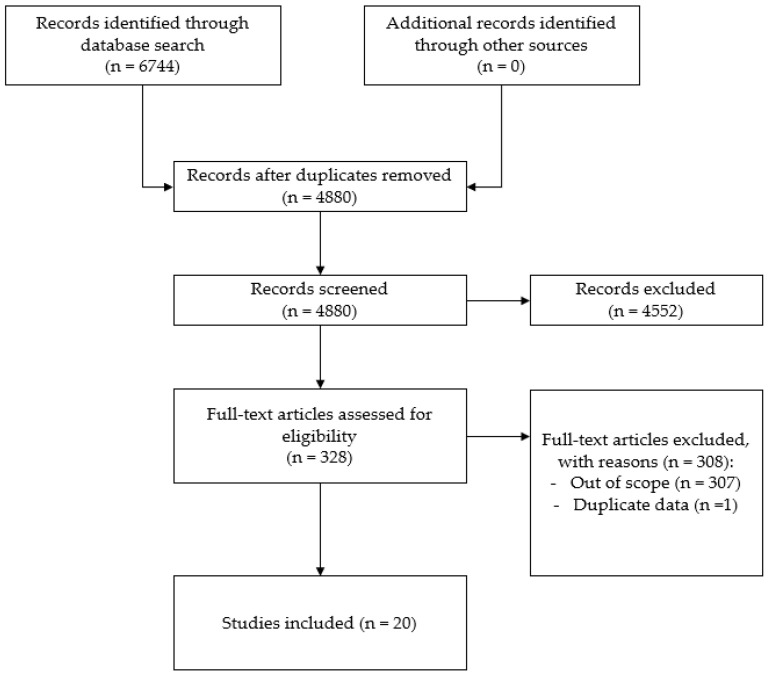
Prisma flow chart.

We identified 6744 studies and, after eliminating 1864 duplicates, we examined 4880 studies. Based on the above inclusion criteria, we selected 328 studies that were considered eligible. We excluded 308 studies because the objective and perceived measures of the environment were used to investigate different characteristics. One article [59] was excluded because it shows the survey result also reported in a more recent article [60] (see Figure 1). Table 1 and Table 2 show the 20 studies included in this review and their characteristics.

The year of publication of the selected studies varies from 2005 to 2019. Their origin is distributed as follows: United States of America (seven studies), Australia (five studies), Canada (one study), China (one study), Ethiopia (one study), Japan (one study), Spain (one study), and Sweden (one study). Two studies used the data collected for the same project (SPOTLIGHT) that was conducted in five European countries (Belgium, France, Hungary, the Netherlands, and the UK). The studies have a sample size ranging from 144 to 24,847 participants. In one study, the sample is made up of women only [32], and one study did not report the gender distribution of the sample [37]. All studies report results from cross-sectional data, except one [31] longitudinal study.

In all studies, PA is measured using a self-report questionnaire: fifteen out of twenty studies (75%) used the International Physical Activities Questionnaire (IPAQ) or its versions; two studies used items from a national survey (Australian National Health Survey and the Behavioral Risk Factor Surveillance System); two studies used a mix of items derived from different questionnaires; one study used one ad hoc item. Only one study [61] also reported objective PA (steps/day) measured with an accelerometer. Considerable variability was observed regarding which kind of PA has been investigated. Some studies investigated general PA without considering intensity, purpose, or kind. At the same time, other studies investigated PA behaviors separately regarding purpose (during leisure time, work, and/or for transportation) or intensity (moderate or vigorous). Otherwise, studies reported leisure-time PA-included walking, leisure-time-excluded walking, active transport (walking and cycling), only walking, or only cycling. The most common PA outcomes were walking (65%) and PA-excluded walking (40%). A total of 40% of the studies reported more than one PA outcome. The majority of the studies (15 out of 20) measured PA using continuous data as min/day (2 studies), min/week (7 studies), day/week (3 studies), or MET*min/week score (3 studies). However, in some of these studies, the data were categorized into three categories (inactive, active, meet recommendation) (2 studies) or dichotomized in “not meet recommendation” and “meet recommendation (3 studies). In the remaining five articles, PA was assessed using a categorical measure such as yes/no (3 studies), no activity/occasionally/frequent (one study), or low-moderate/high (one study).

As regards the environment, all studies investigated the neighborhood’s physical characteristics. However, in some studies, there is no information on how the perceived neighborhood was defined (seven studies). In most studies (60%), it was defined as varying from 1 min to 30 minutes’ walk from own home. Eleven out of twenty studies (55%) used a validated questionnaire as the Neighborhood Environmental Walkability Scale (NEWS) (six studies), the International Physical Activity Prevalence Study’s Environmental Survey Module (IPS) (two studies), the Assessing Levels of Physical Activity and Fitness (ALPHA) questionnaire (two studies), the Multi-Ethnic Study of Atherosclerosis (MESA) survey (one study), and a survey of the perceived availability for green qualities (one study). The remaining nine studies (45%) used ad hoc items. Instead, all studies except one [51] reported the objective definition of the neighborhood. In most studies (70%), it was defined as varying from <300 m to 2 km around the home. In the remaining five studies, it was defined by administrative or census area. Fourteen out of twenty studies (70%) used GIS as an information source, two studies (10%) used Google Street View, two studies (10%) used national archives or tools, and two studies (10%) used mathematics formula (i.e., Euclidean distance) or geography-based algorithms.

Ten characteristics of the neighborhood’s physical environment have been investigated: access/availability of PA/recreational facilities (eight studies), land-use mix (seven studies), street connectivity (six studies), pedestrian facilities (five studies), safe for traffic (four studies), greenness (four studies), aesthetics (four studies), residential density (three studies), bike facilities (three studies), and retail floor-area ratio (one study) (see Table 1). Even if all the studies assessed the environment’s objective and perceived measures, some of them did not report the association between both measures and PA behaviors [30,31,32,60,62]. In particular, the objective environment was used to investigate the difference between urban centers [62] or as a predictor of a city’s development [31] or the perceived environment [49]. Ball et al. [32] reported only agreement between the two environmental measures, and Bourke et al. [30] used a composite score for objective measure (the Walk Score) that is not comparable with the perceived measure.

A total of 75% (six out of eight) of the studies that assess the accessibility of PA facilities reported the association between both types of environment measures and PA behaviors. Three studies reported significantly different PA behaviors in those who perceived better accessibility of PA facilities and no difference with respect to objective availability [63,64,65]. Conversely, two studies reported differences in the PA behavior level only between people with different objective availabilities of PA facilities [33,66]. One study [37] reported any significant effect. The heterogeneity of the results is not explained by the different PA outcomes (recreational PA, leisure-time walk, transportation walk).

A total of 71% (five out of seven) studies that used the land-use mix to measure the physical environment reported an association between both types of environment measures and PA behaviors. Two studies have reported a significant association between both measures of land-use mix and walking [35,64]. However, Sugiyama et al. [67] reported only the association between perceived measure and walking. Kondo et al. [61] reported the same results but only for leisure-time walking in females, whereas no significant association was found for males and transportation walking. At the same time, they reported a significant association with the objective measure and cycling, always only in females. For recreational PA, one study reported a significant association with the perceived measure [34] and one reported no associations [64].

Only three studies (out of seven) reported the association of both measures of street connectivity with PA behaviors. One of them found both associations significant [35], one only found those between objective measure and PA and active travel [68], and the last found no significant association [61]. Also in this case, the heterogeneity of the results is not explained by the different PA outcomes (recreational PA, leisure-time walk, active travel). Three out of four studies investigating the relationship between PA behavior and both measures of pedestrian facilities (quality or availability of sidewalks) reported no significant associations [37,61,69]. The remaining one [64] reported a significant difference between walking (no for recreational PA) levels regarding the objective presence of sidewalks.

Two (out of three) studies that investigated the relationship between traffic safety and PA behaviors reported a significant difference with respect to objective measures [66,68], one of them only in one of the two samples investigated [68]. The remaining one [64] found no differences.

Regarding greenness, two studies reported significant differences in the level of recreational PA with respect to both environmental measures [70,71]. However, the other two studies reported no association for PA [43,64] but a significant difference in transportation activities with respect to the perceived [43] or objective [64] level of greenness.

The two studies investigating aesthetics found no association with recreational PA [34,64]. Conversely, one of them also investigated transportation activity, and it reported a significant difference with respect to both types of environmental measures [64]. Kondo et al. [61] found differences only for leisure-time walking in males regarding perceived aesthetics, whereas no significant association was found for females and transportation walking.

Two studies investigated the effect of residential density on walking behaviors. One of them reported a significant difference for both measures of the environment [35], while the other reported no difference [61].

No differences were found at PA level concerning bike facilities [37,64].

The only study investigating the retail floor-area ratio reported significant predictive power of the perception of this environmental characteristic on walking [35].

Finally, only 30% of the studies (6 out of 20) investigated the relationship between the type of environmental measures [32,35,37,60]. All studies reported poor and no significant agreement or correlation between the perceived and objective physical environment characteristics measures.

Only for the availability of PA facilities are there enough studies to study the possible mediating role of perceived environmental measures in the relationship between objective measures and PA through meta-analytic models. The data were available only for leisure-time physical activity, not walking or active travel. Meta-analysis and MASEM were performed on five studies [33,37,63,64,66] (see Table 3).

### 3.1. Meta-Analysis

The heterogeneity tests computed by the fixed-effect model, performed to calculate the average correlation between PA behaviors and objective availability of PA facilities (Q(4) = 61.75, *p* < 0.001) and between PA behaviors and perceived availability of PA facilities (Q(4) = 9.22, *p* = 0.056), were significant in both cases. Consequently, we ran random-effect models. A significantly low average correlation between the objective availability of PA facilities and PA behavior (r(CI) = 0.152 (0.019–0.280), SE = 0.068) was estimated. The trim-and-fill approach reveals a significant publication bias. The approach estimated the absence of one article with a correlation coefficient above the average effect size estimated. Including this hypothetic article, the average effect size computed is higher (r(CI) = 0.175 (0.051–0.293), SE = 0.064) but still low. A significant but negligible average correlation between the perceived availability and PA (r(CI) = 0.044 (0.023–0.065), SE = 0.011) was estimated. Also in this case, the trim-and-fill approach reveals a significant publication bias. The approach estimated two missing studies with correlation coefficients below the average effect size estimated. Including hypothetical articles, the average effect size computer is lower (r(CI) = 0.037 (0.017–0.052), SE = 0.010).

### 3.2. TSSEM

**Figure 2:**
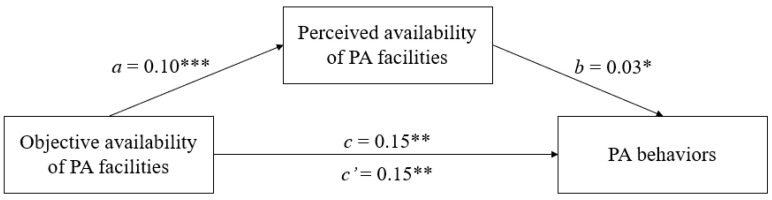
Path diagram depicting the stage-two mediation model of TSSEM. Value is path coefficient. Note. * *p* < 0.05; ** *p* < 0.01; *** *p* < 0.001.

There were no empty cells in the pooled correlation matrix (Table 4). For all studies, correlation between environmental measures and PA were available in the original article or were furnished by the authors (J.D. Mackenbach, personal communication, 7 January 2021). Table 2 shows the pooled correlation matrix computed in the first stage of analysis and used in the second stage. The Q statistic was significant (Q(9) = 69.662, *p* < 0.001), indicating that the five correlation matrices were heterogeneous and justifying the random effect method. Figure 2 shows the path diagram of the model fitted in the second stage. The objective availability of PA facilities significantly predicted the perceived measure (β = 0.10, *p* < 0.001) and PA behavior (β = 0.15, *p* < 0.01). Perceived availability of PA facilities also significantly predicted PA behaviors, but its effect was negligible (β = 0.03, *p* < 0.05). The Sobel test demonstrated that the mediation effect was significant (β = 0.003, *p* < 0.05) but negligible. Indeed, c’ was significant and had the same size as c.

## 4. Discussion

This review summarized the results of 20 previous studies that investigated physical environment characteristics using both perceived and objective measures in relation to PA behaviors. PA behaviors were investigated using different specific outcomes regarding the type of activity (moderate or vigorous exercise, walking, cycling) and purpose (recreational, occupational, transportation). The tendency is to separate PA and walking (for recreative or transportation purposes). Indeed, the most studied outcomes are walking (65% of studies) and PA excluding walking (40%), whereas the less investigated one is PA including walking (15%). Moreover, some authors used guidelines provided by the questionnaire and used the MET*min as the outcome [37,66]. Other authors preferred to use min/week or min/day [34,58] and/or to dichotomize the outcome [30,33].

Ten environmental characteristics were investigated and almost half (9 out of 20) of the studies investigated more than one characteristic. However, only 15 studies reported a relationship between PA behaviors and both types of environmental measures. So, few studies are available for each environment’s characteristics, and the results are heterogeneous. For example, in Coughenour et al. [35] and Hoehner et al. [64], both measures of land-use mix are associated with PA or walking, whereas in Carraca et al. [34] and Sugiyama et al. [67], only the subjective measure is. The small number of studies is also due to the choice to include in this review only the articles that investigated the environmental characteristics by asking the participants to report their perception based on objective indicators such as presence, distance, or number. Studies that investigated personal attitudes towards the environment, i.e., their settled way of thinking or feeling about the environment, were not considered. We suggest including this aspect in future studies because results could be different. As previously noted, the results’ heterogeneity is not explained by the different PA outcomes investigated. For example, walking and recreational PA are both associated with the subjective measure of land-use mix. On the contrary, one PA outcome showed a different pattern of association across studies. We hypothesize that the cultural context could partially explain differences. For example, in our review, we find that greenness has a significant effect on the recreational PA level in European countries [70,71] but not in the U.S.A. [43,64]. People may practice more recreational PA outdoors than indoors in the same countries. Consequently, greenness could be a determinant in the first case, whereas in the second case, the availability of PA facilities could be more fundamental.

The second aim was to investigate the relationship between objective and subjective measures of environmental characteristics in relation to PA. Both measures significantly predict PA behaviors. However, the effect of the perceived measure is negligible and probably lower than that observed (due to publication bias). The indirect effect of the objective measure is also significant but negligible. The significance of the negligible effect is probably due to the amplitude of the sample size. One limit of this study is that it was possible to perform the MASEM only on five studies to make the exposure and outcomes comparable. However, all studies included in the MASEM of this paper have a sample size >100, and the total sample size is very high (*n* = 8936). For this reason, we think that, even if the results should be interpreted carefully, the MASEM presented in the paper could be a valid starting point for further studies. The effect of objective measures seems to be partially mediated by perceived measures, but the mediation effect is very low. So, we conclude that PA behaviors depend on the objective availability of PA facilities and that the perception of people does not explain this effect.

The second interesting result is that the perceived measure is not much predicted by the objective measure. However, few studies have investigated the concordance between measures, and it is not possible to draw a conclusion. The mechanism responsible for this mismatch is still largely unknown. Indeed, it is unclear if people overestimated, believing it is more supportive [37], or underestimated physical environmental characteristics [72]. The reason why people misperceived their environment is not clear. Ball [32] suggested that it could be linked to being or not to being active. Indeed, active women (but not men) reported a lower level of mismatch between measures [32]. Further studies investigating objective measures of physical environment characteristics jointed with subjective measures are needed for at least two reasons. First, the objective measure seems to better explain the variability of PA behaviors than the perceived measure, at least for the availability of PA facilities. However, objective measures could not assess some essential characteristics for the PA [32]. For example, even if PA facilities are available, their quality and/or level of maintenance could determine their use by people. Second, we need to understand why objective and perceived measures are mismatching. It is possible that people do not know their neighborhood, so they are not aware of the presence of the facilities. PA itself could prompt the awareness of the environment. That is, more active people could be more aware of the environment’s characteristics and the opportunities given by the environments [42]. Moreover, reporting PA behaviors, people may implicitly also think of the contextual variables (i.e., environment) and thus remember them more accurately. Indeed, self-reported PA is more consistently related to the neighborhood environment than objectively measured PA [73]. Moreover, it is possible that people considered as available only those facilities which are of good quality. Alternatively, perceived availability could be linked to the cost of PA facilities’ access or use. People possibly do not perceive the availability of the facilities if they cannot afford to spend money for PA. We found only a few studies we considered public (free) and private facilities. Generally, the lower number of studies did not allow us to consider any possible moderator.

## 5. Conclusions

Although more than ten years ago [32], the need for more studies that investigated the characteristics of the physical environment also using objective indicators was underlined, this review highlights a lack of literature development in this direction.

Our study has two principal innovations compared to previous systematic reviews or meta-analyses. First, we included only studies investigating both measures’ type of availability of PA facilities. Second, for the first time, we investigated the relationship between the three variables (PA, objective and perceived availability of PA facilities) in a single model.

Few studies are available for each environment’s characteristics. The most investigated characteristic is the accessibility/availability of PA or recreational facilities, whereas the less investigated characteristic is the retail-floor area ratio. The fact that there are few studies and that there is a big heterogeneity in the results does not allow to draw conclusions on the influence of the physical environment on the probability of being active.

No significant effect was found for the perceived measure on PA. The fact that the objective, but not the perceived environment, was associated with PA suggests that individuals’ level of awareness about their environments may have played a role. This prompts a need to create awareness campaigns, especially amongst subgroups that are more likely to misestimate perceptions.

## Figures and Tables

**Table 1:** List of the 20 selected articles.

Article	Country	*n*	Neighborhood Definition		Physical Activity (PA)	
			Subjective	Objective	Type of PA	Measure of PA
Ball et al., 2008	Australia	1540	2 km from home	2 km from home	(1) PA, (2) walking	yes-no
Borena et al., 2019	Ethiopia	384	n.a.	administrative district	PAw	day/week
Bourke et al., 2018	Australia	228	15 min	suburb	cycling	yes-no
Carraca et al., 2018	5 EU countries *	5205	n.a.	administrative boundaries	PA	min/week
Christian et al., 2013	Australia	1047	<20 min walk	1.6 km	walking	min/week
Coughenour et al., 2019	USA	144	n.a.	census block groups	walking	min/week
Cutumisu and Spence, 2012	Canada	2879	10–15 min walk	1.5 km	(1) PA, (2) walking	MET-min
Dadvand et al., 2016	Spain	3461	10 min walk	<300 m	PA	low-high
de Jong et al., 2012	Sweden	24847	5–10 min walk	<300 m	PA	min/week
Duncan et al., 2010	Australia	2506	from 1 to 30 min (1–5)	census district	walking	min/day
Hoehner et al., 2005	USA	856	<5 min	<400 m	(1) PA, (2) active travel	min/week
Kondo et al., 2009	Japan	156	n.a.	500 m	(1) walking, (2) cycling	min/day
Lee and Moudon, 2008	USA	608	n.a.	1 km	(1) PAw, (2) walking	yes-no
Mackenbach et al., 2018	5 EU countries *	5199	n.a.	n.d.	(1) PA, (2) PAw, (3) walking	min/week
McAlexanderet al., 2012	USA	409	n.a.	800 m	PA	MET-min
McGinn et al., 2007a	USA	1482	20 min walk or 1 mile	1 mile	(1) PAw, (2) walking, (3) active travel	day/week
McGinn et al., 2007b	USA	1482	20 min walk or 1 mile	1 mile	(1) PAw, (2) walking, (3) active travel	day/week
Rodriguez et al., 2008	USA	887	20 min walk or 1 mile	400 m	walking	min/week
Su et al., 2014	China	1343	10–15 min walk	1–1.5 km	(1) PAw, (2) walking	MET-min
Sugiyama et al., 2015	Australia	1412	10–15 min walk	1 km	walking	frequency/week (3 levels)

Note. * Belgium, France, Hungary, the Netherlands, UK. PA = physical activity included walking; PAw = physical activity without walking; active travel = biking and walking for transportation purposes.

**Table 2:** Environmental characteristics investigated in each of the 20 selected articles.

	Aesthetics	Bike Facilities	Greenness	Land Use Mix	PA Facilities	Pedestrian Facilities	Residential Density	Retail Floor-Area Ratio	Safe for Traffic	Street Connectivity
Ball et al., 2008					ü					
Borena et al., 2019					ü					
Bourke et al., 2018										ü
Carraca et al., 2018	ü			ü						
Christian et al., 2013							ü			ü
Coughenour et al., 2019				ü			ü	ü		ü
Cutumisu and Spence, 2012					ü				ü	
Dadvand etal., 2016			ü							
de Jong et al., 2012			ü							
Duncan et al., 2010				ü						
Hoehner et al., 2005	ü	ü	ü	ü	ü	ü			ü	
Kondo et al., 2009	ü			ü		ü	ü			ü
Lee and Moudon, 2008					ü					
Mackenbach et al., 2018					ü					
McAlexanderet al., 2012		ü			ü	ü				
McGinn et al., 2007a									ü	ü
McGinn et al., 2007b			ü							
Rodriguez et al., 2008						ü				
Su et al., 2014	ü	ü		ü	ü	ü			ü	ü
Sugiyama et al., 2015				ü						

**Table 3:** Description of the objective and subjective measure of the availability of PA facilities in the five studies included in the MASEM.

	Objective Measure	Subjective Measure
Borena et al., 2019	Availability (number and type) of PA facilities	Number of recreational facilities
Cutumisu and Spence, 2012	PA facilities in 1500 m	Presence of recreational facilities
Hoehner et al., 2005	Number of recreational facilities	Number of recreational facilities within 5 min walking
Mackenbach et al., 2018	Percentage of street segments in a neighborhood with facilities present	Presence of open recreation areas (park, playing field)
McAlexander et al., 2012	Total number of accessible PA resources	Number of PA resource accessibility

**Table 4:** Pooled correlation coefficients (k = 5, *n* = 8936) for X (objective availability of PA facilities), M (perceived availability of PA facilities), and Y (physical activity). Standard errors are displayed in brackets.

	X	M	Y
X	1		
M	0.104 (0.013) ***	1	
Y	0.044 (0.011) ***	0.149 (0.056) **	1

** *p* < 0.01; *** *p* < 0.001.

## Data Availability

Not applicable.
